# Cost-effectiveness and quality of life of a diet intervention postpartum: 2-year results from a randomized controlled trial

**DOI:** 10.1186/s12889-018-6356-y

**Published:** 2019-01-08

**Authors:** Lars Hagberg, Anna Winkvist, Hilde K Brekke, Fredrik Bertz, Else Hellebö Johansson, Ena Huseinovic

**Affiliations:** 1 Centre for Health Care Science, Faculty of Medicine and Health, Örebro University, Örebro, Sweden; 2 Department of Internal Medicine and Clinical Nutrition, The Sahlgrenska Academy, University of Gothenburg, Box 459, SE-405 30 Gothenburg, Sweden; 3 Department of Nutrition, Institute of Basic Medical Sciences, University of Oslo, Oslo, Norway; 4 Närhälsan, Research and Development Primary Health Care, Region Västra Götaland, Borås, Sweden

**Keywords:** Cost-effectiveness, Quality of life, Weight loss, Postpartum, Primary health care

## Abstract

**Background:**

Pregnancy has been identified as a contributor to obesity. We have shown that a diet intervention postpartum produced a 2-y weight loss of 8%. Here, we present the impact of the diet intervention on cost-effectiveness and explore changes in quality of life (QOL).

**Methods:**

A total of 110 postpartum women with overweight/obesity were randomly assigned to diet (D-group) or control (C-group). D-group received a 12-wk diet intervention within primary health care followed by monthly emails up to the 1-y follow-up. C-group received a brochure. Changes in QOL were measured using the 36-item Short Form Health Survey and EQ-5D. The analysis of cost-effectiveness was a cost-utility analysis with a health care perspective and included costs of intervention for stakeholder, quality-adjusted life-years (QALYs) gained and savings in health care. The likelihood of cost-effectiveness was examined using the net monetary benefit method.

**Results:**

The D-group increased their QOL more than the C-group at 12 wk. and 1 y, with pronounced differences for the dimensions general health and mental health, and the mental component summary score (all *p* < 0.05). Cost per gained QALY was 1704–7889 USD. The likelihood for cost-effectiveness, based on a willingness to pay 50,000 USD per QALY, was 0.77–1.00.

**Conclusions:**

A diet intervention that produced clinically relevant postpartum weight loss also resulted in increased QOL and was cost-effective.

**Trial registration:**

Clinical trials, NCT01949558, 2013-09-24

## Background

Overweight and obesity are growing health problems globally, affecting more than half of the adult population today [1]. Along with the increased risk of adverse health effects and all-cause mortality, obesity has a strong negative impact on health-related quality of life (QOL), which includes the individual’s perception of physical, mental, and social wellbeing [2]. Previous research has reported that nearly all aspects of QOL are adversely affected by elevated body mass index (BMI), and that women with excess weight have lower QOL compared to men of corresponding BMI [3, 4]. In addition, obesity contributes to increased societal costs through both direct health care costs and indirect costs. The latter is a result of decreased years of disability-free life, increased mortality before retirement, early retirement, disability pensions, and reduced productivity [5, 6].

Among women, pregnancy has been identified as an important risk factor for the development and exacerbation of overweight and obesity [7]. This is mainly explained by excessive gestational weight gain and subsequent postpartum weight retention, which increase the risk of complications during succeeding pregnancies [8] and influence long-term maternal health [9, 10]. However, the postpartum period may also spark motivation for lifestyle changes to lose the extra weight gained during pregnancy. Facilitators that converge in this period include increased energy requirement during lactation [11], motivation to return to pre-pregnancy weight [12], desire to serve as a parental role model [13], and an established contact with health care professionals. Also, in Sweden, women can benefit from parental leave until the child is 18 months old old. In addition to the reduced risk of maternal metabolic disease and future pregnancy complications [14], postpartum weight loss may also have an immediate impact on QOL and health care costs [15, 16]. Importantly, increased QOL is a highly relevant patient-centered outcome and an essential component in cost-effectiveness analyses. However, data on the long-term effect of postpartum lifestyle interventions on QOL and cost-effectiveness are missing, especially in real world settings. This information is critical to guide politicians and financers involved in decision-making processes about resource allocation.

We have recently conducted an effectiveness trial to evaluate whether a 12-wk diet intervention can produce weight loss among postpartum women with overweight and obesity within a primary health care setting in Sweden. The results showed that women randomized to diet intervention achieved a greater weight loss after 12 wk. (6.1 vs 1.6 kg, *p* < 0.001) and 1 y (10.0 vs 4.3 kg, *p* = 0.004) compared to the control group [17]. When women with a new pregnancy between 1 and 2 y were excluded, an effect emerged also at 2 y (8.2 vs 4.6 kg, *p* = 0.038) [18]. In this report, we evaluate the cost-effectiveness of the diet intervention and explore changes in QOL, as compared to a control group, in postpartum women with overweight/obesity within the context of primary health care in Sweden.

## Methods

### Subjects and study design

The LEVA (Lifestyle for Effective Weight loss during Lactation) in Real Life study was a two-arm randomized controlled trial evaluating the effectiveness of a 12-wk diet intervention in producing weight loss among postpartum women within the primary health care setting in Sweden. Details on the study procedures, the statistical power calculations, and the primary outcome in regard to weight were reported previously [17]. In brief, women with a self-reported BMI ≥27 kg/m^2^ in early postpartum were enrolled during March 2012–October 2014 in the Gothenburg area. In total, 110 women entered the trial at 6–15 wk. postpartum for baseline measurements and group allocation. Women were randomized to diet group (D-group, *n* = 54) or control group (C-group, *n* = 56). Follow-up visits were performed 12 wk., 1 y and 2 y after baseline. The trial was approved by the regional ethical committee in Gothenburg and written informed consent was obtained from all women.

### Study groups

Women randomly assigned to the D-group met with the dietitian for 1.5 h of structured individual diet behavior modification treatment. The aim of the diet treatment was to achieve a reduction of daily energy intake by 500 kcal in order to achieve a weekly loss of 0.5 kg and a final loss of 6 kg after 12 wk. The diet plan was based on the Nordic Nutrition Recommendations 2004 [19] and consisted of four key dietary principles to be implemented one at a time [20]. During the intervention, bi-weekly standardized cell phone text messages were sent to women in the D-group to ask for their body weight and to provide personalized feedback. After wk. 6 of the intervention, women received a telephone call to allow for questions and more thorough feedback. From wk. 12 to 1 y, the D-group received monthly information/reminder emails and were asked to report body weight and provided with personalized feedback. The C-group was given a brochure on healthy eating and was not provided with any further material. No follow-up contact was provided to either group between 1 and 2 y.

### Anthropometric measurements

Body weight was measured using an electronic scale [21], with women wearing light clothing. Height was measured via a wall-mounted stadiometer. Pre-pregnancy BMI was calculated as self-reported pre-pregnancy weight divided by the square of measured height. Gestational weight gain was obtained by self-report.

### QOL measurements

**Fig. 1:**
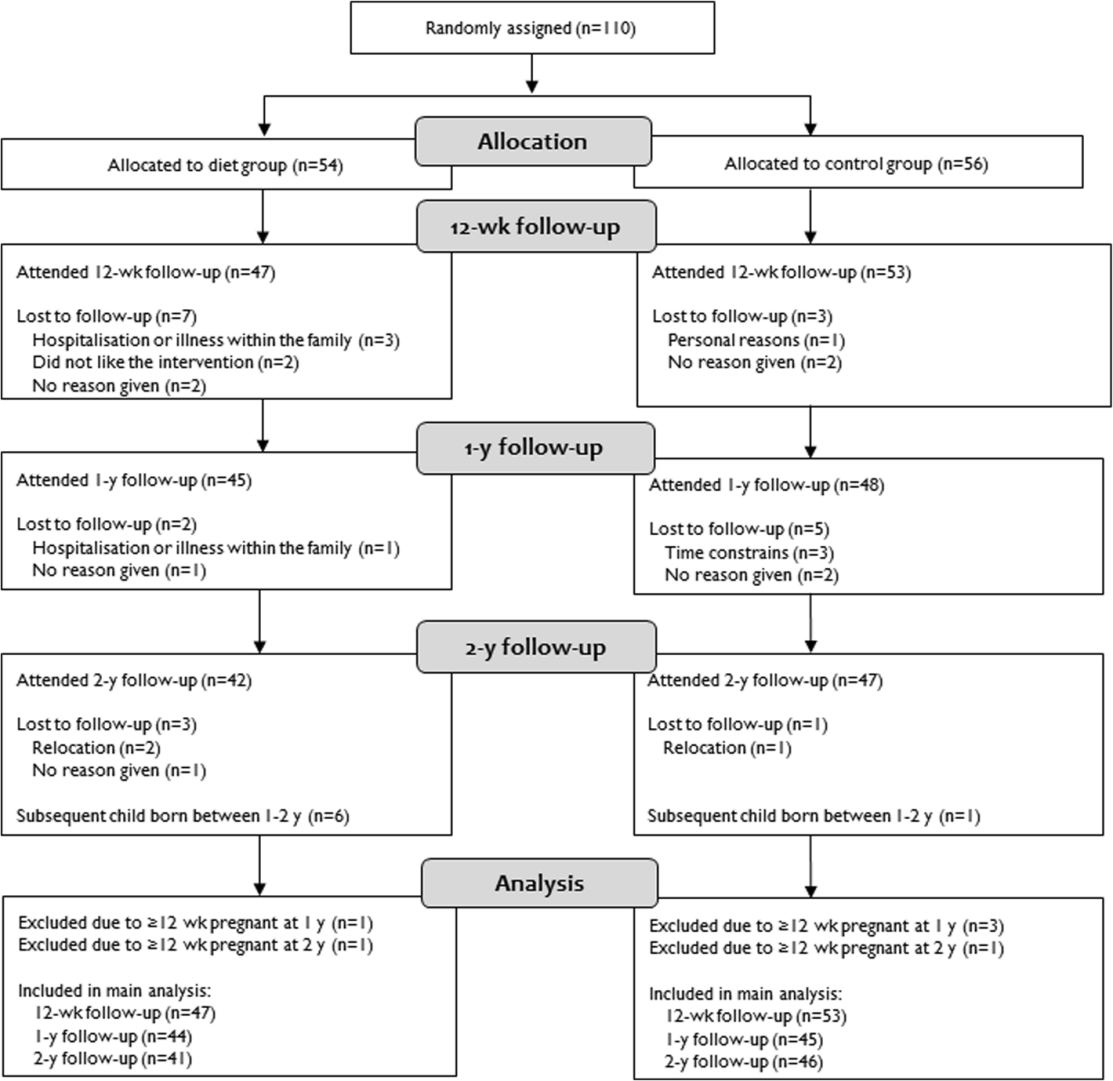
Flow chart of study participants in the LEVA in Real Life trial

QOL was measured using the 36-item Short Form Health Survey (SF-36 RAND), the EuroQol 5D (EQ-5D-3 L), and the EuroQol Visual Analog Scale (EQ-VAS). The SF-36 RAND consists of 36 questions grouped into eight dimensions: physical functioning, limitations in physical role functioning, bodily pain, general health, vitality, social functioning, limitations in emotional role functioning, and mental health [22]. Each dimension is scored from 0 (worst imaginable health) to 100 (best imaginable health). The SF-36 RAND also includes a physical component summary score and mental component summary score [22]. From SF-36 RAND, we derived the SF-6D score. It is based on 11 questions in the SF-36 RAND questionnaire and consists of six dimensions [23, 24]. The EQ-5D-3 L is a self-classifier with measures of five dimensions: mobility, self-care, usual activities, pain/discomfort, and anxiety/depression [25]. A score was computed based on a value tariff from a British population [26]. The EQ-VAS is a measure of overall health status on a 20-cm line graded from 0 (worst imaginable health) to 100 (best imaginable health). In the QOL-analysis, women pregnant > 12 week gestation at a follow-up visit were excluded as QOL can be affected by new pregnancies (Fig. 1).

### Health economic analysis

The analysis of cost-effectiveness was a cost-utility analysis from a health care system perspective with a 2 y time horizon. All women were included throughout all time points (*n* = 110). Cost-effectiveness ratios were expressed as cost per gained quality-adjusted life-years (QALY). QALY was estimated based on SF-6D, EQ-5D-3 L and EQ-VAS scores. Costs of intervention for stakeholder, QALY, and savings in health care use were included, but not the cost for participants or changes in production. Production losses were not expected as this was a healthy study population, and the women were initially were on maternal leave.

Swedish krona (SEK) have been converted to USD based on the price 9.0 SEK = 1.0 USD. Costs were expressed in 2017 price levels, and recalculated using the Swedish consumer price index [27]. No research costs or costs of method development were considered when calculating changes in QALY, and costs were discounted 3% in the second year. Gains in QALY were calculated by utilizing the difference in change in QOL between the D- and C-group and the length of time. Gains in QALY were assumed to develop linearly over time. For example, in comparison to the control group, if the increase in QOL is 0.08 at 3 months and 0.16 at 1 year, the mean change during the first 3 months is 0.04 (i.e., (0.00 + 0.08)/2), and during the next 9 months 0.12 (i.e., (0.08 + 0.16)/2). Altogether, the QALY gain for this year would be 0.10 (i.e., (0.04 × 3/12) + (0.12 × 9/12)).

The uncertainty due to variance in the trial data was handled using the net monetary benefit (NMB) method [28], where QALYs are replaced by the amount of money decision makers are willing to pay for a QALY. By repeatedly drawing a random sample with replacement, a scatter plot was created of 5000 bootstrapped incremental cost-effectiveness ratios. Individual values were used for savings in health care use and QALY, and mean values were used for costs in the two study groups. This produced estimates of the likelihood that the diet intervention was cost-effective compared to the C-group using 0–100,000 USD as thresholds of willingness to pay for a QALY [29]. Results are presented in a cost-effectiveness acceptability curve [30]. Besides analyses of the impact of variances in data, a sensitivity analysis was performed for the impact of higher intervention costs and lower gain in QALY.

### Statistical analysis

Missing data were replaced using three different imputation methods: multiple imputation (primary analysis for QOL), single imputation using the third quartile value (sensitivity analysis for QOL) and stochastic imputation (primary analysis for the cost-effectiveness analysis). The multiple imputation procedure used linear regression analysis and the multivariate imputation by chained equations method. This procedure generated 20 complete data sets. The model included variables related to outcome, and/or to drop out, including study group, age, parity, BMI, gestational weight gain, and QOL at all study visits. Furthermore, single imputation was used to replace missing data with systematically unfavorable values representing the group-specific third quartile change value. Finally, stochastic imputation was used to replace missing values in the cost-effectiveness analysis due to the need for individual data for QALYs and health care in the NMB analysis. This model included the same variables as in the multiple imputation.

Student’s t-test was used to examine differences in changes in QOL between the D- and C-group. Effect size was estimated and classified as low (0.20), medium (0.50), and high (0.80) according to Cohen’s classification [31]. Analyses were performed using SPSS software version 22 (IBM), and SAS software version 9.4 (SAS Institute Inc). Statistical significance was considered at *p* < 0.05 (two-sided).

## Results

### Subjects

Of the 110 randomized women, 100 (91%), 93 (85%) and 89 (81%) completed the 12-wk, 1-y, and 2-y follow-up, respectively (Fig. 1). There were no statistically significant differences between the two groups for the presented background characteristic in Table 1.

**Table 1:** Baseline characteristics of the study participants in the LEVA in Real Life trial

Variable	All women (*n* = 110)	Diet group (*n* = 54)	Control group (*n* = 56)	*P*-value
Age, y	32.2 ± 4.6	31.8 ± 4.5	32.6 ± 4.7	0.357
Parity, n	2.0 (1.0; 2.0)	2.0 (1.0; 2.3)	2.0 (1.0; 2.0)	0.128
Pre-pregnancy BMI, kg/m^2^	28.4 (26.0; 32.4)	27.4 (25.4; 32.3)	28.8 (26.8; 33.0)	0.121
BMI at baseline, kg/m^2^	31.0 (28.8; 33.6)	30.7 (28.6; 34.1)	31.2 (28.8; 33.5)	0.995
Gestational weight gain^a^, kg	17.4 ± 7.4	18.2 ± 6.9	16.5 ± 7.7	0.246
Education, No. (%)				0.164
Short education at high school	1 (1)	1 (2)	0 (0)	
≤ 3 y beyond high school	43 (39)	25 (46)	18 (32)	
> 3 y beyond high school	66 (60)	28 (52)	38 (68)	
Marital status, No. (%)				0.239
Married or cohabitant	108 (98)	52 (96)	56 (100)	
Single	2 (2)	2 (4)	0 (0)	
Lactation status, No. (%)				0.059
None	18 (16)	10 (19)	8 (14)	
Partial	29 (26)	19 (35)	10 (18)	
Exclusive	63 (57)	25 (46)	38 (68)	

Data are mean ± SD for normally distributed variables, median (1^st^; 3^rd^ quartile) for non-normally distributed variables and No. (%) for categorical variables. ^a^Based on self-reported weight

### QOL

Greater improvements in QOL were observed in the D-group than in the C-group after 12 wk. in the EQ-5D-3 L (*p* = 0.03) and after both 12 wk. and 1 y in the EQ-VAS (*p* = 0.002 and p = 0.03, respectively). No difference was observed in the SF-6D score (Table 2). As for the eight dimensions in the SF-36 RAND, pronounced differences were shown for the dimensions general health and mental health, and for the mental component summary score at 12 wk. and 1 y, with greater improvements in the D-group than in the C-group (all *p* < 0.05, effect size ranging from 0.44–0.69). There was a general decrease in QOL within both groups from 1 to 2 y. As a result there were no between-group differences at 2 y, except for the difference in general health that remained (*p* = 0.02, Table 2).

**Table 2:** Changes in quality of life in the LEVA in Real Life trial with missing values imputed using multiple imputation^a^

	Diet group	Control group	Difference diet vs control group^b^	*P*-value	Effect size^c^
SF-6D					
Baseline	0.676	0.660			
Change after 12 wk.	0.043 (0.106)	0.029 (0.098)	0.015 (− 0.256; 0.055)	0.47	0.14
Change after 1 y	0.072 (0.098)	0.054 (0.098)	0.018 (− 0.023; 0.059)	0.38	0.18
Change after 2 y	0.028 (0.117)	0.024 (0.101)	0.005 (−0.040; 0.049)	0.84	0.04
EQ-5D-3 L					
Baseline	0.832	0.838			
Change after 12 wk	0.041 (0.105)	−0.035 (0.213)	0.075 (0.009; 0.142)	0.03	0.45
Change after 1 y	0.042 (0.141)	0.004 (0.150)	0.038 (−0.023; 0.099)	0.21	0.26
Change after 2 y	−0.016 (0.221)	−0.015 (0.171)	− 0.001 (− 0.082; 0.080)	0.98	0.01
EQ-VAS					
Baseline	65.6	70.3			
Change after 12 wk	10.9 (16.4)	0.2 (18.1)	10.7 (3.9; 17.5)	0.002	0.62
Change after 1 y	12.1 (14.8)	4.9 (16.5)	7.1 (0.6; 13.7)	0.03	0.46
Change after 2 y	6.5 (19.6)	2.0 (15.3)	4.5 (−3.0; 12.1)	0.23	0.26
SF-36 Physical functioning					
Baseline	87.5	87.6			
Change after 12 wk	2.4 (15.6)	2.2 (11.6)	0.2 (−5.2; 5.5)	0.94	0.01
Change after 1 y	6.2 (11.0)	4.1 (10.8)	2.1 (−2.4; 6.6)	0.35	0.19
Change after 2 y	3.9 (14.2)	1.4 (13.2)	2.5 (−3.1; 8.0)	0.38	0.18
Limitations in physical role functioning					
Baseline	67.6	61.6			
Change after 12 wk	10.1 (37.9)	20.3 (39.3)	−10.2 (−25.0; 4.6)	0.17	−0.26
Change after 1 y	18.4 (40.1)	17.9 (43.1)	0.5 (−16.1; 17.0)	0.95	0.01
Change after 2 y	14.2 (40.4)	11.4 (46.3)	2.8 (−14.9; 20.5)	0.76	0.06
Bodily pain					
Baseline	71.4	67.9			
Change after 12 wk	1.2 (24.8)	4.8 (24.0)	−3.6 (−13.0; 5.9)	0.46	−0.15
Change after 1 y	9.4 (26.8)	12.0 (27.5)	−2.5 (−13.4; 8.3)	0.64	−0.10
Change after 2 y	7.1 (25.9)	8.9 (26.4)	−1.8 (− 12.2; 8.6)	0.73	−0.07
General health					
Baseline	70.0	75.8			
Change after 12 wk	7.9 (15.6)	−2.9 (15.9)	10.8 (4.5; 17.1)	< 0.001	0.69
Change after 1 y	10.4 (16.1)	−0.5 (18.6)	11.0 (3.6; 18.3)	0.004	0.63
Change after 2 y	6.0 (16.3)	−2.9 (18.5)	8.9 (1.4; 16.4)	0.02	0.51
Vitality					
Baseline	51.3	52.6			
Change after 12 wk	10.1 (19.2)	1.2 (19.0)	9.0 (1.4; 16.5)	0.02	0.47
Change after 1 y	10.7 (24.9)	6.9 (21.3)	3.8 (−5.8; 13.5)	0.43	0.16
Change after 2 y	3.7 (22.7)	−2.0 (23.9)	5.7 (−4.0; 15.3)	0.25	0.24
Social functioning					
Baseline	80.3	81.3			
Change after 12 wk	7.8 (24.9)	5.5 (16.1)	2.2 (−5.9; 10.3)	0.58	0.11
Change after 1 y	9.3 (23.8)	4.5 (19.9)	4.9 (−3.8; 13.6)	0.27	0.22
Change after 2 y	3.6 (28.2)	−0.4 (19.6)	4.0 (−5.7; 13.7)	0.41	0.16
Limitations in emotional role functioning					
Baseline	77.8	78.6			
Change after 12 wk	7.0 (39.4)	−0.6 (39.0)	7.7 (−7.4; 22.7)	0.32	0.19
Change after 1 y	9.9 (40.2)	−2.7 (46.7)	12.6 (−4.6; 29.8)	0.15	0.29
Change after 2 y	−7.8 (47.0)	−15.0 (45.6)	7.2 (−11.4; 25.9)	0.44	0.16
Mental health					
Baseline	77.3	78.9			
Change after 12 wk	4.8 (12.5)	−2.9 (13.4)	7.8 (2.6; 12.9)	0.004	0.59
Change after 1 y	4.4 (13.3)	−3.1 (14.0)	7.5 (1.5; 13.5)	0.02	0.55
Change after 2 y	−2.3 (17.9)	−5.9 (15.8)	3.6 (−3.4; 10.6)	0.31	0.21
Physical component summary score					
Baseline	47.5	46.8			
Change after 12 wk	2.2 (7.9)	3.7 (7.3)	−1.5 (−4.5; 1.5)	0.32	−0.20
Change after 1 y	5.1 (8.8)	5.4 (9.2)	−0.3 (−4.0; 3.4)	0.87	−0.03
Change after 2 y	5.1 (9.8)	4.1 (9.8)	1.0 (−3.1; 5.1)	0.63	0.10
Mental component summary score					
Baseline	45.6	46.7			
Change after 12 wk	3.7 (10.2)	−1.3 (9.0)	5.0 (1.3; 8.8)	0.009	0.52
Change after 1 y	3.3 (11.0)	−1.7 (11.8)	5.0 (0.4; 9.6)	0.03	0.44
Change after 2 y	−1.8 (13.6)	−5.3 (12.9)	3.5 (−2.2; 9.1)	0.22	0.26

Abbreviations: *SF-6D* 6-dimensional short-form 6D, *EQ-5D-3 L* The EuroQol 5D, *EQ-VAS* The EuroQol visual analog scale, and *SF-36* 36-item short form health survey. Values are mean (SD) if not indicated differently. ^a^The multiple imputation procedure used linear regression analysis and the multivariate imputation by chained equations method, and generated 20 complete data sets. The model included variables that were related to the outcome, and/or related to drop out, including study group, age, parity, BMI, gestational weight gain, and QOL at all study visits. Numbers of women at baseline, 12 wk., 1 y and 2 y were 110, 100 (47 and 53 women in the diet and control groups, respectively), 89 (44 and 45, respectively) and 87 at 2 y (41 and 46, respectively) after exclusion of women pregnant > 12 week at a follow-up visit. Drop-outs are presented in Fig. 1 and missing data were limited and missing at random. ^b^ Mean (95% CI). ^c^ Effect size calculated according to Cohen [31] where a change of 0.2–0.5 is considered small, 0.5–0.8 is considered moderate and > 0.8 is considered large

When missing data were replaced with the group-specific third quartile value, all between-group differences were maintained (data not shown). The only statistically significant difference that did emerge between the D- and C-groups was the change in “limitation in emotional role functioning” that became significant at both 1 y (9.3 vs − 9.5, *p* = 0.02) and 2 y (− 2.5 vs − 20.8, *p* = 0.02).

### Cost-effectiveness

**Fig. 2:**
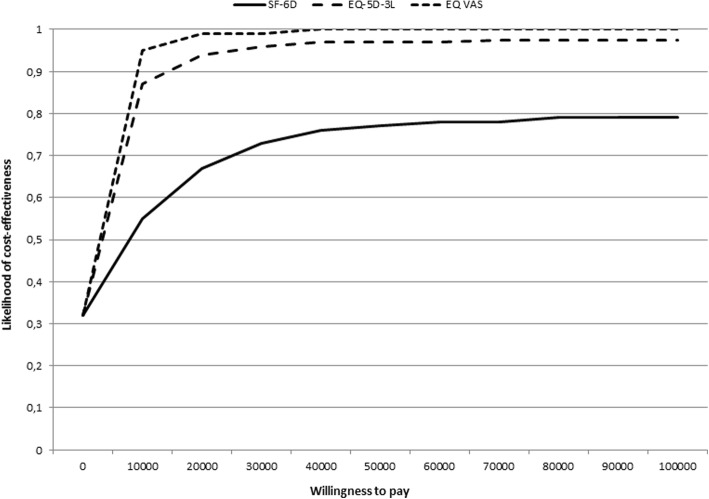
Likelihood of cost-effectiveness when willingness to pay for a quality-adjusted life-year was 0–100,000 USD in the LEVA in Real Life trial. The analysis is based on the three quality of life-instruments Short-Form 6D (SF-6D), the EuroQol 5D (EQ-5D-3 L), and the EuroQol-VAS (EQ-VAS)

Over the 2 yrs., the increase in QALY for the D-group compared to the C-group was 0.03 (*p* = 0.38) using SF-6D, 0.09 (*p* = 0.05) using EQ-5D-3 L and 0.13 (*p* = 0.006) using EQ-VAS. The cost of the D-group was 229 USD per participant and the cost of the C-group was 5 USD per participant (Table 3). During the trial, the costs for health care use increased by 599 USD in the D-group and by 611 USD in the C-group (*p* = 0.63). Cost per gained QALY was 7889 USD based on SF-6D; 2367 USD based on EQ-5D-3 L; and 1704 USD based on EQ-VAS. The likelihood of cost-effectiveness was 0.77, 0.97 and 1.00, respectively, when willingness to pay for a QALY was set to 50,000 USD (Fig. 2). In the sensitivity analysis, with doubled cost of the intervention and halved gain in QALY, the cost per QALY was 7000–32,000 USD.

**Table 3:** Cost-effectiveness expressed in cost per quality-adjusted life-year (QALY). All costs are per participant and in USD. Costs of health care use are changes from baseline

Cost-effectiveness	Diet group *n* = 54	Control group *n* = 56	Diet vs. control group
Intervention costs			
Start of intervention, meeting with dietician (1 h 50 min)	87	–	87
Telephone costs, time (40 min) and telephone fee	32	–	32
Email conversation, time (1 h 20 min)	63	–	63
Equipment (loan of balance)	7	–	7
Cost of printed material	2	4	−2
Sum of direct costs	191	4	187
Overhead, administration and local costs, 20%	38	1	37
Sum of total costs per woman	229	5	224
Cost of health care use	599	611	−12
QALY			
QALY based on SF-6D	0.088	0.061	0.027
QALY based on EQ-5D-3 L	0.069	−0.021	0.090
QALY based on EQ-VAS	0.188	0.063	0.125
Cost-effectiveness			
Cost per QALY based on SF-6D			7889
Cost per QALY based on EQ-5D-3 L			2367
Cost per QALY based on EQ-VAS			1704

## Discussion

We set out to evaluate the impact of a primary health care-based diet intervention on QOL and cost-effectiveness in a postpartum weight loss trial. We found that the D-group improved their QOL more at 12 wk. and 1 y, and had a greater increase for the dimensions general health and mental health, and the mental component summary score, than did the C-group. Medium effect sizes were reached according to Cohen’s classification [31]. Furthermore, the cost per QALY was 7889 USD (SF-6D), 2367 USD (EQ-5D-3 L) and 1704 USD (EQ-VAS). With a willingness to pay 50,000 USD for a QALY, the likelihood of cost-effectiveness was 0.77–1.00. To the best of our knowledge, this is the first effectiveness trial to demonstrate that a diet intervention postpartum performed in real life settings improved QOL and was cost-effective. This is in addition to the favorable effects previously reported on anthropometric outcomes [17].

Previous research among non-pregnant individuals with obesity has reported that weight loss is associated with an increase in physical health but not in mental health [32]. In the current trial, there was no difference between study groups in the physical component summary score; however, a between-group difference was observed for the mental component summary score at 12 wk. and 1 y. This finding could be related to fewer physical limitations (excluding recovery from childbearing) in this young female study population (mean age 32.2 y) with relatively low BMI in the context of weight loss trials (pre-pregnancy BMI 28.4 kg/m^2^, hence within the overweight range). Furthermore, although recovery from childbearing and being on maternal leave are plausible reasons for the improved QOL within both study groups, the explanation the greater improvement in the D-group is probably multifactorial. For example, the increase in QOL in the D-group could be related to the weight loss per se, and/or to the feeling of being able to control one’s lifestyle and weight. In addition, success in adhering to the new dietary regime, reaching pre-pregnancy weight and the design of the diet intervention may also have played a role. Nevertheless, between 1 and 2 y, there was a general decrease in QOL for both groups, in line with results from other lifestyle interventions [33, 34].

In the present trial, the decline in QOL and mental health from 1 to 2 y could be related to regaining weight between 1 and 2 y, indicating decreased compliance with the diet regime. The decrease in QOL could also be associated with returning to work or studies, as most women in Sweden stay at home from between 1 to 2 years. Hence, the transition to “normal life” may have impacted their QOL and also made it more challenging to maintain lifestyle habits established during parental leave. They were thus facing new barriers to weight management. Novel strategies and additional support might be needed to maintain weight loss and QOL in working life as compared to achieving improvements during parental leave. These strategies could include knowledge about stress management and the addition of physical activity, which has been shown to increase both physical and mental aspects of QOL [16, 35]. In sum, our results indicate that diet interventions postpartum should be supplemented with further efforts related to returning to working life.

The estimated costs per gained QALY (1704–7889 USD) for the diet intervention can be considered low. There are, however, no formal thresholds for what is considered good cost-effectiveness. What can be prioritized on a restricted budget depends on a number of factors besides cost-effectiveness, including affordability, budget impact, fairness, and feasibility. WHO argues that a threshold should simply be seen as an indication of poor, good, or very good value for money [36]. Despite this, there is ongoing discussion of what cost-effectiveness ratios are usually accepted. Cost-effectiveness ratios of 50,000–100,000 USD in USA [29] and 32,000–50,000 USD in UK [29] have often been accepted by stakeholders in Sweden and other Western countries. Thus, the cost-effectiveness ratios of the current trial can be considered low in relation to what Western countries are willing to pay for a QALY, and the likelihood of cost-effectiveness is high. Although there are no general recommendations for the threshold for the likelihood of cost-effectiveness for a change in routine care, there are arguments that it should be close to 0.50 [37]. Sensitivity analyses, in which higher costs and a lower gain in QALY were modeled, did not change the assumption of cost-effectiveness. This strengthens the conclusion that the diet intervention can be considered cost-effective.

The diet intervention in the LEVA in Real Life trial was developed and evaluated in a previous efficacy trial, the LEVA trial [38], conducted by our research group. In that trial, the diet intervention produced a weight loss of 9% after 12 wk. that was maintained at 10% after 1 y. In the related cost-utility analysis [39], the diet intervention was found to be cost-effective, with a cost per gained QALY of 8643–9785 USD and a likelihood of cost-effectiveness of 0.87–0.93. Compared to the LEVA trial, the present diet intervention was less costly (incremental cost 225 USD vs 303 USD). In the LEVA in Real Life trial, the diet intervention was somewhat cheaper but had approximately similar effects. However, it is important to note that it was performed in an ordinary health care setting. As far as we know, there are no other cost-effectiveness studies of diet intervention for postpartum women with overweight or obesity. In addition, diet interventions in other patient groups confirm that diet interventions for individuals with overweight or obesity are in general cost-effective [40].

This trial has several strengths. First, the diet intervention was delivered and evaluated within ordinary primary health care in Sweden; thus, the effect and costs should be representative of those involved in running the diet treatment in routine care. Nevertheless, future research should examine the effect of postpartum diet treatment in countries with less generous parental leave policies. Second, QOL can be viewed from different dimensions. In Sweden, SF-36 is the most established QOL-instrument, and has the advantage of providing information on multiple dimensions of QOL. Specific changes in the different dimensions of QOL represent an interesting additional aspect beyond what is provided by regular health economic QOL instruments that show QOL in relation to only a single dimension. Third, there is always a degree of uncertainty when estimating QALY based on a QOL instrument. It can be asked whether the questionnaires capture the actual change of QOL, whether the preference valuation is made by a relevant group of individuals and which of the methods of preference valuation (time trade-off, standard gamble, or rating scale) is used. We used three different QOL instruments in which the preferences were evaluated by a general population as well as by the affected patients. We also used all three valuation methods. Thus we should have reduced the uncertainty involved in estimating QALY. The conclusion is the same regardless of which QOL instrument was used. Further confirmation is provided by the fact that similar results were found in the previous LEVA trial, where the same diet intervention was evaluated. Finally, the cost-effectiveness analysis is based on real life data, thereby minimizing the need for assumptions.

The present report also has some limitations. Although attrition was low compared to other weight loss [41, 42] and postpartum [7, 43] trials, the women who dropped out during the trial may differ systematically from those who remained. Therefore, to reduce the risk of overestimating the effect, several intention-to-treat analyses were performed using different imputation strategies [44]. All models generated the same result, strengthening and confirming the conclusions from this trial. Note, however, that due to the explorative approach used to examine the dimensions of QOL, *p*-values should be interpreted with caution.

## Conclusion

A diet intervention that produced clinically relevant postpartum weight loss also resulted in increase in QOL and was cost-effective. Maternity leave usually entails changes in everyday life, and so this period provides a unique opportunity for health care to offer lifestyle treatment and promote postpartum weight loss through dietary changes.

## Abbreviations

BMI: Body mass index
C-group: Control group
D-group: Diet group
EQ-5D-3 L: EuroQol 5D
EQ-VAS: EuroQol Visual Analog Scale
LEVA: Lifestyle for Effective Weight loss during Lactation
NMB: Net monetary benefit
QALY: Quality-adjusted life-years
QOL: Quality of life
SF-36 RAND: 36-Item Short Form Health Survey
USD: US dollars
